# Preoperative Sinonasal Computed Tomography Score in Chronic Rhinosinusitis with Nasal Polyps

**DOI:** 10.3390/tomography8010007

**Published:** 2022-01-04

**Authors:** Giuseppe Brescia, Giacomo Contro, Alessandra Ruaro, Anna Chiara Frigo, Umberto Barion, Gino Marioni

**Affiliations:** 1 Department of Neuroscience DNS, Otolaryngology Section, University of Padova, 35128 Padova, Italy; giuseppe.brescia@aopd.veneto.it (G.B.); giacomo.contro@gmail.com (G.C.); alessandra.ruaro.1@studenti.unipd.it (A.R.); umberto.barion@aopd.veneto.it (U.B.); 2 Department of Cardiac-Thoracic-Vascular Sciences and Public Health, University of Padova, 35128 Padova, Italy; annachiara.frigo@unipd.it

**Keywords:** chronic rhinosinusitis with nasal polyps, CRSwNPs, computed tomography staging system, Lund-Mackay score, endotype

## Abstract

This study investigated the relationship between sinonasal inflammatory involvement according to the computed tomography (CT) staging system (Lund–Mackay score) with clinical, laboratory, histopathological and prognostic features of chronic rhinosinusitis with nasal polyps (CRSwNP). Seventy-eight patients with CRSwNP who had undergone surgery were enrolled. Total (*p* = 0.0062), ethmoid (*p* = 0.0496), sphenoid (*p* = 0.0335), ostiomeatal complex (OMC) (*p* = 0.0235) and frontal (*p* = 0.0164) CT scores were predictive of non-steroidal anti-inflammatory drugs-exacerbated respiratory disease (NERD) in the univariate analysis. Total (*p* = 0.0022), ethmoid (*p* = 0.0290), sphenoid (*p* = 0.0370), frontal (*p* = 0.0116), maxillary (*p* = 0.0357) and OMC (*p* = 0.0058) CT scores were predictve of asthma at the univariate analysis. No significant differences were found between patients with vs. without allergy in terms of total and partial CT scores. High blood eosinophil counts (>0.24 vs. ≤0.24 cells × 10^9^/L) resulted in being associated with total (*p* = 0.0213), maxillary (*p* = 0.0227) and ethmoid (*p* = 0.0491) CT scores in the univariate analysis. Higher ethmoid (*p* = 0.0006) and total sinonasal (*p* = 0.0027) CT scores were found to predict histopathologically eosinophil CRSwNPs in the univariate analysis. CT scores did not result as predictive of NSAID-exacerbated respiratory disease, asthma, or blood eosinophil count at the multivariate analysis. Risk of relapse was related to the presence of NERD (*p* = 0.0207, HR [95% CI] 3.914 [1.232–12.435]), higher preoperative total (HR = 1.098 95%CI: 1.001–1.204, *p* = 0.0486) and frontal sinus CT scores (HR = 1.555 95%CI: 1.006–1.886, *p* = 0.0218), but these results were not confirmed by the multivariable analysis. Sinonasal CT scores showed significant differences in this heterogeneous inflammatory condition. Identifying CRSwNP characteristics is necessary to avoid generic treatments with poor outcomes.

## 1. Introduction

Chronic rhinosinusitis (CRS) is divided into two phenotype-based groups according to the presence (CRSwNPs) or absence of nasal polyps. This is definitely an overly simple classification given that CRS appears clinico-pathologically to be a very heterogeneous inflammatory condition. Currently, rhinologists consider that CRSwNP is no longer a “*unicum*” but a group of several sub-types with different patho-physiological features, the so-called endotypes. Several studies have stratified nasal polyposis based on histologic features, inflammatory factors, and different circulating inflammatory cells [1,2]. Each endotype differs in disease recurrence risk and requires targeted follow-up protocols and post-operative treatments [3].

Computed tomography (CT) is the most common imaging modality in CRSwNPs for the evaluation of inflammatory sinus mucosal disease [4,5]. Because of CT high spatial resolution and ability to depict fine osseous detail, it is the imaging test of choice for providing the necessary information for endoscopic sinus surgery (ESS) planning, such as the presence of fluid and degree of mucosal thickening, the presence of bone dehiscence or osteitis, and the anatomy of the sinuses, including important sinonasal anatomic variants [6]. The most widely applied CT staging system of sinonasal inflammatory changes is the Lund–Mackay score (LMS) [7,8]. In very recent years, CT scores have received further attention as potential hallmarks for recognizing CRSwNPs phenotypes, endotypes, and prognosis [7,9,10]. Mamat Nasir et al. [11] found that sinus CT score correlated with the symptom score for both eosinophilic and non-eosinophilic CRSwNP. Furthermore, in a cohort of patients with CRS, olfactory test resulted as correlated negatively with the overall and ipsilateral LMS [12].

The main aim of this study was to investigate the relationship between sinonasal inflammatory involvement according to LMS with clinical, laboratory, histopathological and prognostic features of CRSwNPs patients who underwent ESS because of medical treatment failure. Possible associations between the aforementioned phenotyping and endotyping features and any single paranasal subsite (maxillary, ethmoid, frontal and sphenoid) or ostiomeatal complex (OMC) involved at CT scan in CRSwNPs patients were also evaluated. CRSwNPs are distinct entities, in histological appearance and genetic and protein expression patterns, we herein hypothesized a potential role of CT score to differentiate CRSwNPs sub-types. The results of this investigation could highlight a role of preoperative CT not only in the surgical planning but also in the rationalization of follow up and post-operative treatment.

## 2. Materials and Methods

### 2.1. Patients

The study was conducted in accordance with the principles of the Helsinki Declaration. All patients signed a detailed informed consent form regarding the processing and publication of their data. Data were examined in agreement with the Italian privacy and sensitive data laws, and the internal regulations of Padova University’s Otolaryngology Section.

The study retrospectively evaluated 78 adult patients suffering from CRSwNPs: 50 patients were males and 28 were females with a mean age of 48.9 ± 12.5 years (median age 49 years). Conventional medical therapies had failed and patients, therefore, underwent ESS between 2015 and 2019 for at least an endoscopic grade II polyposis [13]. For the purposes of the investigation, patients with sinonasal polyps associated to systemic diseases (e.g., eosinophilic granulomatosis with polyangiitis, granulomatosis with polyangiitis, sarcoidosis, primary ciliary dyskinesia, cystic fibrosis) were excluded. 

All patients underwent a preoperative CT. The CT scans were acquired with a 64-multislice scanner using 0.6–1.0 mm slice thickness, 120 Kv, and 80–160 mAs; moreover, sagittal and coronal reconstructions were always included. The CT findings were analyzed and staged according to the Lund and Mackay [8] staging system by an experienced rhinologist and sinonasal surgeon (G.B.). In order to evaluate the extent of the chronic inflammatory process, a score from 0 to 2 was assigned to each sinus (maxillary, anterior ethmoid, posterior ethmoid, sphenoid and frontal) depending on the grade of their opacification. For the OMC region the score was 0 or 2 (not occluded/occluded). The score ranged from 0 (no abnormalities of any sinonasal structure) to 24 (total opacification of all considered sinonasal structures).

Patients’ clinical features were collected. Information about hypersensitivity to acetylsalicylic acid or other non-steroidal anti-inflammatory drugs (NSAIDs) were obtained from patients’ medical histories recorded in Padova University Hospital’s electronic archives (Galileo). The diagnosis of asthma was confirmed according to the definition of the Global Initiative on Asthma [14]. The whole study population underwent a pneumological visit and pulmonary function tests (spirometry). Based on the clinical and functional situation, when appropriate, the pulmonologist indicated that the following tests should be performed: reversibility tests (in the case of airway obstruction) or hyper-reactivity tests (in the absence of airway obstruction). All patients underwent preoperative tests at the Laboratory Medicine Service of Padova University Hospital regarding total and specific IgE for the main inhalant allergens. All patients had a blood sample taken approximately 1 month before surgery also to obtain their eosinophil count.

After ESS, surgical tissue was stained with hematoxylin and eosin to consider the eosinophil count, examining five high-power fields (5HPF) (400×) selected from each sample and recording the average number of eosinophils. The histopathological diagnosis of eosinophil CRSwNPs corresponded to a mean score higher than 10 eosinophils/5HPF.

All patients were treated postoperatively with isotonic saline solution irrigations twice a day (20 mL per irrigation), nasal steroids (mometasone furoate 200 µg daily [100 µg per nostril], or fluticasone furoate 110 µg daily [55 µg per nostril]). According to the EPOS guidelines [4], intranasal steroid treatment began after the first postoperative outpatient check-up (in the present series after a median value of 8 days after surgery). Adequate therapy was prescribed for asthmatic and allergic patients.

Follow-up with rigid 0° or 30° endoscopes was scheduled at 3, 6 and 12 months after ESS, and yearly thereafter. Patients were classified as cases of recurrence if they had endoscopic evidence of at least grade I polyposis [13]. Grade II or higher CRSwNP recurrent cases or symptomatic patients for more than 3 months unresponsive to medical therapy underwent sinonasal CT during follow-up.

### 2.2. Statistical Analysis

Statistics were conducted on 78 patients except for the relapse occurrence analysis which was conducted on 75 patients (3 patients were lost at follow-up).

Data were analysed with SAS 9.4 (SAS Institute Inc., Cary, NC, USA) for Windows.

The normality of sinonasal sub-site and total CT scores was inspected graphically with a Q-Q plot and Shapiro–Wilk test. The normality assumption was refused when *p* < 0.10.

Sinonasal sub-site and total CT scores predictivity of non-steroidal anti-inflammatory drug-exacerbated respiratory disease (NERD), asthma, allergy, blood eosinophils count (≤0.24 cells × 10^9^/L vs. >0.24 cells × 10^9^/L) [2], and histological eosinophil features were analysed with univariate logistic regression. The CT sub-site scores resulted statistically significant at the 5% level, were introduced in a multivariable logistic regression model. The results are presented as Wald *p* value, odds-ratio (OR) estimates and 95% confidence interval (CI). Odds-ratios and 95% CI are expressed per unit of score increase. The linearity assumptions were evaluated with the Hosmer and Lemeshow goodness-of-fit test. The total CT score was not considered in the multivariate analysis, since it is composed by the sub-site scores. Multicollinearity of the sub-site scores was evaluated with Spearman correlation coefficient and variance inflation factor (VIF). Since VIF resulted lower than 2, multicollinearity was judged to be not present. 

The predictivity of clinical, laboratory and sinonasal sub-site and total CT scores respectful time to recurrence was analysed with univariate Cox regression analysis. The results are expressed as Wald *p* value, hazard ratio estimates and 95% CI. Hazard ratios and 95% CI are expressed per unit of score increase for quantitative variables. The proportionality assumption was verified with the Kolmogorov-type supremum test through 1000 replications. Area under the curve (AUC) and 95% CI were calculated at selected time-points (6, 12, 18, 24, 30, 36, 42 and 48 months) for predictive accuracy evaluation of CT scores. The variables that resulted statistically significant at the 5% level in the univariate analysis were considered in a multivariable Cox regression model.

The considered CRSwNP cohort was characterized by a median follow period of 22 months (mean 26.4 ±18.6 months) and by a median disease-free interval of 22 months (mean 24.7 ±16.9 months).

## 3. Results

The associations between radiological findings and (i) clinical, (ii) laboratory, (iii) histopathological and (iv) prognostic features of our patients’ cohort are reported.

### 3.1. Sinonasal Computed Tomography (CT) Scores and Clinical Features

**Figure 1:**
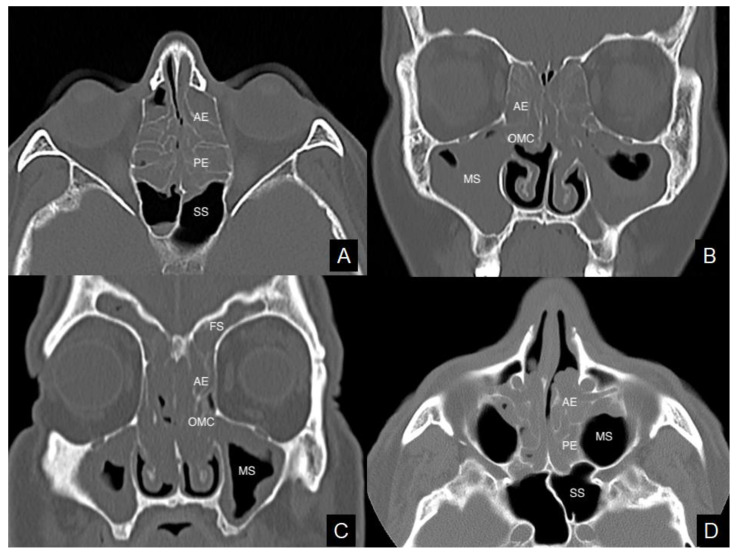
Axial and coronal views of computed tomography imaging. Massive and homogeneous sinus opacification in a chronic rhinosinusitis with nasal polyps (CRSwNPs) patient with non-steroidal anti-inflammatory drugs-exacerbated respiratory disease (NERD) (**A,B**). Ethmoid inflammatory involvement in a CRSwNPs case with asthma (**C**). Polyps occupying the central compartments in a patient with eosinophil CRSwNPs (**D**). AE = anterior ethmoid; MS = maxillary sinus; OMC = ostiomeatal complex; PE = posterior ethmoid; SS = sphenoidal sinus. FS = frontal sinus.

Table 1 and Table 2 summarize mean and median values of the total CT score and of each sinonasal sub-site in CRSwNPs patients with NERD and asthma, respectively. At the univariate logistic regression analysis increasing ethmoid (OR = 1.358, *p* = 0.0496), sphenoid (OR = 1.722, *p* = 0.0335), OMC (OR = 1.706, *p* = 0.0235) and frontal (OR = 1.742, *p* = 0.0164) CT scores resulted predicting the presence of NERD (Figure 1A,B). The result was confirmed considering the total CT score (OR = 1.193, *p* = 0.0062). At the multivariable analysis the sub-site CT score resulted anymore statistically significant.

The total sinonasal CT score was significantly higher in asthmatic patients than in non-asthmatic ones (OR = 1.141, *p* = 0.0022). In particular, ethmoid (OR = 1.223, *p* = 0.0290) (Figure 1C), sphenoid (OR = 1.582, *p* = 0.0370), frontal (OR = 1.522, *p* = 0.0116), maxillary (OR = 1.643, *p* = 0.0357) and OMC (OR = 1.505, *p* = 0.0058) CT scores resulted predictive of asthma at the univariate analysis, but not at the multivariate. 

No significant differences were found between patients with vs. without allergy (33 vs. 45 cases, respectively) in terms of total CT scores and partial ones according to the different sinonasal sub-sites.

### 3.2. Sinonasal CT Scores and Laboratory Results

Table 3 reports mean and median values of the total CT scores and of any sinonasal sub-site in patients with blood eosinophil count ≤0.24 cells × 10^9^/L vs. >0.24 cells × 10^9^/L. This categorization was performed according to the cut-off values reported by Brescia et al. [2]. At univariate analysis total (OR = 1.106, *p* = 0.0213), maxillary (OR = 1.729, *p* = 0.0227) and ethmoid (OR = 1.200, *p* = 0.0491) predicted blood eosinophil counts >0.24 vs. ≤0.24 cells × 10^9^/L. No differences between the two sub-cohorts were found regarding OMC and frontal sinus.

### 3.3. Sinonasal CT Scores and Histopathological Evidence 

Higher ethmoid (OR = 1.399, *p* = 0.0006) and total sinonasal (OR = 1.142, *p* = 0.0027) CT scores were found in the sub-cohort of histopathologically eosinophil CRSwNPs (Figure 1D) than in the non-eosinophil ones. The whole quantitative analysis is reported in Table 4. 

### 3.4. CT Scores and Prognosis after Endoscopic Sinus Surgery (ESS)

Twelve out of 75 patients (16%) developed CRSwNPs relapse after ESS. At univariate Cox regression analysis, risk of disease recurrence in CRSwNPs patients was associated with presence of NERD (*p* = 0.0207, HR [95% CI] 3.914 [1.232–12.435]), pre-operative total and frontal sinus CT scores (*p* = 0.0486, HR [95% CI] 1.098 [1.001–1.204] and *p* = 0.0218, HR [95% CI] 1.555 [1.066–2.267], respectively). The CT scores of the other sinonasal sub-sites were not predictive of CRSwNPs recurrence.

The predictive accuracy of the frontal sinus CT scores resulted higher than that of the total score at all selected time-points, except at 12 and 18 months, although with overlapping CIs (Table 5).

Since total and frontal sinus CT scores are associated, in the multivariable model we considered NERD and frontal CT scores that resulted anymore associated with disease recurrence (NERD: *p* = 0.2605, HR [95% CI] 2.213 [0.555–8.829; frontal CT score: *p* = 0.1640 HR [95% CI] 1.367 [0.880–2.124]).

## 4. Discussion

The main aim of this study was to investigate the associations between sinonasal inflammatory involvement according to Lund and Mackay [8] staging system and clinical, laboratory, histopathological and prognostic features of CRSwNPs patients. Possible associations between the aforementioned CRSwNPs types and any single sinonasal sub-site involved (maxillary, ethmoid, frontal, or sphenoid) and OMC at CT scan were also evaluated. It was preferred to analyze the role of the CT staging system in relation to clinical and prognostic objective aspects of CRSwNPs instead of considering subjective symptomatological aspects as recently done by other authors [15,16]. The possibility of using preoperative partial CT scores as an additional instrument to stratify CRSwNP sub-types is nowadays worthy of investigation. The main strengths of the investigation lie in the homogeneity of the series of patients considered since: (i) only cases of CRSwNPs were considered; (ii) histopathological analyses were all undertaken by a dedicated head and neck pathologist; (iii) ESS was performed by the same team of surgeons; (iv) the endoscopic follow-up after surgery was conducted by the same team; (v) recurrent CRSwNPs was always confirmed endoscopically. The main weaknesses of the study concern the retrospective setting and limited number of patients.

### 4.1. Sinonasal CT Staging System and Prognosis in Chronic Rhinosinusitis with Nasal Polyps (CRSwNPs)

LMS has previously been used as an index to predict recurrence of CRSwNPs. In 2019, Kim et al. [17] considered 134 CRSwNPs patients who had undergone ESS: they found that high LMS were associated with worse disease control in eosinophil-type CRSwNPs but not in non-eosinophil ones. Meng et al. [18] recruited a total of 272 consecutive CRSwNPs patients who had undergone ESS. The authors [18] determined the anterior ethmoid sinus CT score (AE score), posterior ethmoid sinus score (PE score) and maxillary sinus score (M score), then calculated E/M ratio (ratio between total of the AE and PE scores for both sides and M score for both sides). The E/M ratio was significantly higher in the recurrence group; E/M ratio showed high accuracy as a predictor for CRSwNPs recurrence [18]. Considering our series, total and frontal sinus CT scores according to Lund and Mackay [8] staging system were significantly higher in patients who relapsed after surgery.

### 4.2. Sinonasal CT Staging System and Phenotype/Endotype Features in CRSwNPs

Studying 100 cases of CRS (28 CRSwNPs and 72 CRSsNPs), Kwun et al. [19] found that LMS was significantly higher in CRSwNPs cases and in the aspirin nasal provocation test-positive ones. In the present series, ethmoid, sphenoid, frontal, OMC and total CT scores were all significantly higher in patients with NERD than in those without. Moreover, in our group of patients, total sinonasal CT score was significantly higher in CRSwNPs patients with asthma than in those without. In particular, ethmoid, sphenoid, frontal, maxillary and OMC pre-operative CT scores were significantly higher in the asthmatics. No significant differences were found between patients with vs. without allergy in terms of total and partial pre-operative CT scores according to the different sinonasal sub-sites. 

Recently, attention has turned to the value of blood sampling and inflammatory cell assays in shedding light on the patho-physiology of CRSwNPs and predicting the course of the disease [20]. Eosinophil levels in peripheral blood have been investigated as a potential predictor of a diagnosis of eosinophil CRSwNPs at histology [21]. Analyzing the relationship between pre-operative sinonasal CT scores and blood eosinophil count in our series, a significant difference emerged between patients with blood eosinophil counts ≤0.24 cells × 10^9^/L and >0.24 cells × 10^9^/L in terms of maxillary, ethmoid, and total CT scores (see Table 3).

As is well known, in CRSwNPs the eosinophil histotype has been extensively studied and found to be associated with the prognosis for CRSwNPs: eosinophil CRSwNPs resulted in being associated with higher recurrence rates and shorter disease-free intervals after treatment than non-eosinophil forms [22]. It has been suggested that eosinophil and non-eosinophil CRSwNPs are two distinct entities, in both histological appearance and genetic and protein expression patterns [23,24]. Considering the role of pre-operative CT scores in this specific setting, Meng et al. [25] analyzed 200 consecutive CRSwNPs patients (123 eosinophil CRSwNPs and 77 non-eosinophil ones). They found that E/M ratio was significantly higher in histopathologically eosinophil CRSwNP and concluded that E/M ratio had a high predictive value in diagnosis of this CRSwNPs sub-type. Evaluating 38 and 14 eosinophil and non-eosinophil CRSwNPs cases respectively on the basis of histopathological examination, Rai et al. [26] found that E/M ratio and total sinonasal CT score were the most useful surrogate markers for preoperative differentiation of eosinophil and non-eosinophil CRSwNPs. In our series, higher ethmoid and total sinonasal CT scores were found in the sub-cohort of histopathologically eosinophil CRSwNPs than in the non-eosinophil ones. Interestingly, no significant differences were disclosed in terms of E/M ratio between eosinophil and non-eosinophil CRSwNPs (quantitative data not reported).

In summary, analyzing the diagnostic and prognostic utility of total and partial CT scores was particularly relevant with regard to asthmatics, NERD and histopathologically eosinophil CRSwNPs. This finding can be explained by an increased aggressiveness of these CRSwNPs types, in terms of widespread involvement of sinuses and nasal cavities and a tendency to relapse. If this preliminary information were confirmed by studies with larger, preferably prospective, case series, it would be advisable to plan closer follow-ups and dedicated post-operative medical therapies for CRSwNP patients at greater risk of relapse. Otherwise, it is interesting to note that we did not find the same result for allergic CRSwNPs: further studies must be conducted to thoroughly understand the mechanisms and clinical course in allergic CRSwNPs. Central compartment atopic disease (CCAD) has recently been described as a variant of CRSwNPs that is significantly associated with allergy [27]. On CT, CCAD patients showed an involvement mainly of the nasal cavities [7]. Generally speaking, our results appeared to be in opposition to the hypotheses that the allergic form of CRSwNPs would mainly affect the so-called “central compartment” and, on the contrary, asthmatic, NERD and eosinophil CRSwNPs develop in the paranasal sinuses and then extend to the nasal cavities. Our preliminary CT-based results support the hypothesis that asthmatic, NERD and eosinophil CRSwNPs are not compartmental inflammatory diseases but affect the entire sinonasal mucosa

## 5. Conclusions

As hypothesized, CT scores confirmed the presence of significant differences within CRSwNPs, a clinico-pathologically very heterogeneous inflammatory condition. In particular, in a univariate setting, CT scores were significantly different comparing asthmatics, NERD and pathologically eosinophil CRSwNPs patients. The fact that CT scores were not predictive of NSAID-exacerbated respiratory disease, asthma, or blood eosinophil count in our multivariate model could depend on the correlation between CT scores and on the limited number of cases considered. In univariate analysis, the risk of CRSwNP relapse resulted in being related to NERD, higher preoperative total and frontal sinus CT scores, but these results were not confirmed by the multivariable analysis, probably due to the low sample size and number of recurrences. The preoperative sinonasal CT variables considered herein should be further investigated for their potential as a valuable aid in providing patients with correct information about prognosis and, mainly, to avoid generic treatments with poor outcomes.

## Figures and Tables

**Table 1:** Summary statistics, univariate and multivariate logistic regression analysis of any single sinonasal sub-site and total computed tomography (CT) scores predicting NERD (yes vs. no) in CRSwNPs patients.

	NERD		Univariate Logistic Regression		Multivariate Logistic Regression	
No. Cases = 78	No (No. Cases = 68)	Yes (No. Cases = 10)	*p* Value	OR (95% CI)	*p* Value	OR (95% CI)
OMC CT score						
Mean (SD)	1.60 (1.63)	3.00 (1.70)	0.0235	1.706 (1.075–2.709)	0.2592	1.381 (0.788–2.419)
Median (IQR)	2.00 (0.00–3.00)	4.00 (2.00–4.00)				
Frontal CT score						
Mean (SD)	1.22 (1.40)	2.50 (1.58)	0.0164	1.742 (1.107–2.742)	0.3434	1.333 (0.735–2.419)
Median (IQR)	1.00 (0.00–2.00)	2.50 (2.00–4.00)				
Maxillary CT score						
Mean (SD)	2.07 (1.07)	2.80 (1.03)	0.0548	2.034 (0.985–4.199)	NC	NC
Median (IQR)	2.00 (1.00–3.00)	3.00 (3.00–3.00)				
Ethmoid CT score *						
Mean (SD)	4.49 (2.66)	6.40 (2.80)	0.0496	1.358 (1.000–1.842)	0.7974	1.054 (0.704–1.580)
Median (IQR)	4.00 (2.00–7.00)	8.00 (4.00–8.00)				
Sphenoid CT score						
Mean (SD)	0.54 (0.94)	1.40 (1.84)	0.0335	1.722 (1.043–2.841)	0.5947	1.174 (0.651–2.116)
Median (IQR)	0.00 (0.00–1.00)	0.00 (0.00–3.00)				
Total CT score						
Mean (SD)	9.93 (5.56)	16.10 (7.00)	0.0062	1.193 (1.051–1.354)	NC	NC
Median (IQR)	9.00 (6.00–14.00)	19.00 (9.00–22.00)				

*: anterior plus posterior ethmoidal scores; CI: confidence interval; IQR: interquartile range; NC: not considered (according to Statistical Analysis Methods); NERD: non-steroidal anti-inflammatory drug (NSAID)-exacerbated respiratory disease; OR: odds-ratio; SD: standard deviation.

**Table 2:** Summary statistics, univariate and multivariate logistic regression analysis of any single sinonasal sub-site and total CT scores in CRSwNPs patients with vs. without asthma.

	Asthma		Univariate Logistic Regression		Multivariate Logistic Regression	
No. Cases = 78	No (No. Cases = 48)	Yes (No. Cases = 30)	*p* Value	OR (95% CI)	*p* Value	OR (95% CI)
OMC CT score						
Mean (SD)	1.35 (1.49)	2.47 (1.80)	0.0058	1.505 (1.125–2.012)	0.1392	1.300 (0.918–1.842)
Median (IQR)	1.50 (0.00–2.00)	4.00 (0.00–4.00)				
Frontal CT score						
Mean (SD)	1.04 (1.25)	1.93 (1.66)	0.0116	1.522 (1.099–2.108)	0.4218	1.198 (0.770–1.864)
Median (IQR)	1.00 (0.00–2.00)	2.00 (0.00–4.00)				
Maxillary CT score						
Mean (SD)	1.96 (0.97)	2.50 (1.20)	0.0357	1.643 (1.034–2.612)	0.3003	1.312 (0.785–2.192)
Median (IQR)	2.00 (1.00–2.50)	3.00 (2.00–3.00)				
Ethmoid CT score *						
Mean (SD)	4.19 (2.73)	5.60 (2.57)	0.0290	1.223 (1.021–1.466)	0.8921	1.017 (0.798–1.296)
Median (IQR)	4.00 (2.00–6.50)	6.00 (4.00–8.00)				
Sphenoid CT score						
Mean (SD)	0.44 (0.85)	1.00 (1.39)	0.0370	1.582 (1.028–2.434)	0.7068	1.104 (0.660–1.846)
Median (IQR)	0.00 (0.00–0.50)	0.00 (0.00–2.00)				
Total CT score						
Mean (SD)	8.98 (5.08)	13.50 (6.56)	0.0022	1.141 (1.049–1.242)	NC	NC
Median (IQR)	8.00 (5.00–13.50)	13.50 (7.00–20.00)				

*: anterior plus posterior ethmoidal scores; NC: not considered (according to Statistical Analysis Methods); SD: standard deviation; IQR: interquartile range.

**Table 3:** Summary statistics, univariate and multivariate logistic regression analysis of any single sinonasal sub-site and total CT scores in patients with blood eosinophil count ≤0.24 cells × 10^9^/L vs. >0.24 cells × 10^9^/L.

	Blood Eosinophil Count (Cells × 10^9^/L)		Univariate Logistic Regression		Multivariate Logistic Regression	
No. Cases = 75	≤0.24 Cells × 10^9^/L * (No. Cases = 29)	>0.24 Cells × 10^9^/L (No. Cases = 46)	*p* Value	OR (95% CI)	*p* Value	OR (95% CI)
OMC CT score						
Mean (SD)	1.48 (1.57)	2.04 (1.76)	0.1654	1.221 (0.921–1.620)	NC	NC
Median (IQR)	2.00 (0.00–2.00)	2.00 (0.00–4.00)				
Frontal CT score						
Mean (SD)	1.14 (1.25)	1.59 (1.61)	0.2047	1.238 (0.890–1.721)	NC	NC
Median (IQR)	1.00 (0.00–2.00)	1.00 (0.00–3.00)				
Maxillary CT score						
Mean (SD)	1.79 (0.98)	2.39 (1.08)	0.0227	1.729 (1.080–2.769)	0.0700	1.574 (0.964–2.573)
Median (IQR)	2.00 (1.00–2.00)	3.00 (2.00–3.00)				
Ethmoid CT score **						
Mean (SD)	4.10 (2.51)	5.37 (2.69)	0.0491	1.200 (1.001–1.439)	0.1790	1.140 (0.942–1.381)
Median (IQR)	4.00 (2.00–5.00)	6.00 (4.00–8.00)				
Sphenoid CT score						
Mean (SD)	0.34 (0.94)	0.87 (1.20)	0.0611	1.654 (0.977–2.802)	NC	NC
Median (IQR)	0.00 (0.00–0.00)	0.00 (0.00–2.00)				
Total CT score						
Mean (SD)	8.86 (5.00)	12.26 (6.38)	0.0213	1.106 (1.015–1.206)	NC	NC
Median (IQR)	9.00 (6.00–11.00)	13.00 (7.00–18.00)				

*: blood eosinophil count cut-off according to Brescia et al. [2]; **: anterior plus posterior ethmoidal scores; CI: confidence interval; IQR: interquartile range; NC: not considered (according to Statistical Analysis Methods); OR: odds-ratio; SD: standard deviation.

**Table 4:** Summary statistics and univariate logistic regression analysis of any single sinonasal sub-site and total CT scores in patients with histopathologically eosinophil vs. non-eosinophil CRSwNPs.

	Histologically Eosinophil CRSwNPs		Univariate Logistic Regression	
No. Cases = 78	No (No. Cases = 36)	Yes (No. Cases = 42)	*p* Value	OR (95% CI)
OMC CT score				
Mean (SD)	1.39 (1.71)	2.12 (1.63)	0.0594	1.303 (0.990 1.715)
Median (IQR)	0.00 (0.00–3.00)	2.00 (0.00–4.00)		
Frontal CT score				
Mean (SD)	1.11 (1.39)	1.62 (1.53)	0.1324	1.274 (0.929 1.748)
Median (IQR)	0.50 (0.00–2.00)	1.00 (0.00–3.00)		
Maxillary CT score				
Mean (SD)	1.94 (0.92)	2.36 (1.19)	0.0973	1.439 (0.936 2.214)
Median (IQR)	2.00 (1.00–3.00)	2.50 (2.00–3.00)		
Ethmoid CT score *				
Mean (SD)	3.53 (2.78)	5.76 (2.26)	0.0006	1.399 (1.154 1.696)
Median (IQR)	3.00 (1.50–5.50)	6.00 (4.00–8.00)		
Sphenoid CT score				
Mean (SD)	0.42 (0.94)	0.86 (1.22)	0.0902	1.483 (0.940 2.338)
Median (IQR)	0.00 (0.00–0.00)	0.00 (0.00–2.00)		
Total CT score				
Mean (SD)	8.39 (5.95)	12.71 (5.50)	0.0027	1.142 (1.047 1.245)
Median (IQR)	6.50 (3.50–12.00)	12.50 (8.00–18.00)		

*: anterior plus posterior ethmoidal scores; SD: standard deviation; IQR: interquartile range.

**Table 5:** Area under the curve (AUC), standard error and 95% confidence interval (CI) from the Cox regression model at selected follow-up time-points.

	Frontal CT score		Total CT score	
Follow-Up (Months)	AUC (Standard Error)	95% Confidence Interval	AUC (Standard Error)	95% Confidence Interval
6	0.6694 (0.0579)	0.5558–0.7829	0.629 (0.0785)	0.4751–0.7830
12	0.4131 (0.1422)	0.1345–0.6918	0.5411 (0.0980)	0.3489–0.7332
18	0.5167 (0.1286)	0.2647–0.7688	0.5389 (0.0883)	0.3658–0.7121
24	0.6476 (0.0959)	0.4596–0.8356	0.6073 (0.1087)	0.3943–0.8202
30	0.7104 (0.1015)	0.5115–0.9093	0.6458 (0.1094)	0.4313–0.8602
36	0.7885 (0.0988)	0.5948–0.9821	0.7475 (0.0972)	0.5571–0.9380
42	0.7977 (0.0989)	0.6039–0.9915	0.7588 (0.0971)	0.5686–0.9491
48	0.9759 (0.0279)	0.9213–1.0000	0.8355 (0.1517)	0.5382–1.0000

## Data Availability

The data presented in this study are available on request from the corresponding author.
